# Local Extinction and Unintentional Rewilding of Bighorn Sheep (*Ovis canadensis*) on a Desert Island

**DOI:** 10.1371/journal.pone.0091358

**Published:** 2014-03-19

**Authors:** Benjamin T. Wilder, Julio L. Betancourt, Clinton W. Epps, Rachel S. Crowhurst, Jim I. Mead, Exequiel Ezcurra

**Affiliations:** 1 Department of Botany and Plant Sciences, University of California Riverside, Riverside, California, United States of America; 2 National Research Program, Water Mission Area, U.S. Geological Survey, Reston, Virginia, United States of America; 3 Department of Fisheries and Wildlife, Oregon State University, Corvallis, Oregon, United States of America; 4 Department of Geosciences, and Sundquist Center of Excellence in Paleontology, East Tennessee University, Johnson City, Tennessee, United States of America; 5 University of California Institute for Mexico and the United States (UC MEXUS), Riverside, California, United States of America; University of York, United Kingdom

## Abstract

Bighorn sheep (*Ovis canadensis*) were not known to live on Tiburón Island, the largest island in the Gulf of California and Mexico, prior to the surprisingly successful introduction of 20 individuals as a conservation measure in 1975. Today, a stable island population of ∼500 sheep supports limited big game hunting and restocking of depleted areas on the Mexican mainland. We discovered fossil dung morphologically similar to that of bighorn sheep in a dung mat deposit from Mojet Cave, in the mountains of Tiburón Island. To determine the origin of this cave deposit we compared pellet shape to fecal pellets of other large mammals, and extracted DNA to sequence mitochondrial DNA fragments at the 12S ribosomal RNA and control regions. The fossil dung was ^14^C-dated to 1476–1632 calendar years before present and was confirmed as bighorn sheep by morphological and ancient DNA (*a*DNA) analysis. 12S sequences closely or exactly matched known bighorn sheep sequences; control region sequences exactly matched a haplotype described in desert bighorn sheep populations in southwest Arizona and southern California and showed subtle differentiation from the extant Tiburón population. Native desert bighorn sheep previously colonized this land-bridge island, most likely during the Pleistocene, when lower sea levels connected Tiburón to the mainland. They were extirpated sometime in the last ∼1500 years, probably due to inherent dynamics of isolated populations, prolonged drought, and (or) human overkill. The reintroduced population is vulnerable to similar extinction risks. The discovery presented here refutes conventional wisdom that bighorn sheep are not native to Tiburón Island, and establishes its recent introduction as an example of unintentional rewilding, defined here as the introduction of a species without knowledge that it was once native and has since gone locally extinct.

## Introduction

As recorded in *Cmiique Iitom* —the language of the Seri people, an indigenous community of the coast of Sonora, Mexico and nearby Tiburón Island— Orion's belt, *Hapj*, consists of three stars. The middle star represents the mule deer, *hap*, and the two flanking stars are bighorn sheep, *mojet*, and pronghorn antelope, *haamoja*. When the great hunter of the sky, *Azoj Cmiique* (Scorpius), fired his arrow, it struck *hap* but missed the others. After dripping onto Tiburón Island, the mule deer's blood remained in the sky as the red star *Azoj haait* (Alpha or Betelgeuse). For the Seri, this myth explains why mule deer, but not bighorn sheep or pronghorn antelope, historically inhabited the island [1].

The events that have led to the formation of modern ecosystems, especially extinctions, are often cryptic in occurrence and causation. The anomalous absence of species in either the fossil record or on modern landscapes raises several questions. Did particular species once occur that are now lost? If so, what caused their extinctions, and are they reversible? How do we establish biological baselines to determine conservation priorities and strategies in the absence of historical data?

For example, controversial rewilding efforts to restore and even resurrect lost megafauna [2]–[5] at the very least demand accurate baselines. In this paper, we coin the term “unintentional rewilding” to mean the introduction of a species, deliberate or otherwise, without knowledge that it was once native and has since gone locally extinct. Here are a couple of examples. In North America, European horses were introduced during the Spanish conquest, subsequently went feral, and unwittingly replaced native horses that were genetically the same but became extinct at the end of the Pleistocene [6], [7]. European domesticated horses gone wild are regarded by federal land management agencies in the USA as an exotic species that is harmful to native wildlife habitat and thus should be eradicated or reduced in numbers. Accepting these feral horses as native would challenge current management mandates within the federal government.

A second example involves bison. Extreme drought at the start of the twenty-first century drove bison from the adjacent Kaibab Plateau, where they were introduced and bred with cattle during the 1930s into Grand Canyon National Park, where bison are now trampling riparian areas and archeological sites. Holocene evidence for bison is scant in the Grand Canyon, and the modern herd may include bison-cattle hybrids, bolstering the National Park Service's case for removal. But what if the modern herd contains no hybrids and future paleontological evidence shows that bison occupied the Grand Canyon intermittently throughout the Holocene? Awareness of cases such as the North American horses and Grand Canyon bison will surely increase with expanded paleoecological studies and advances in genomics [8], [9], and will continue to raise fundamental questions about conservation targets and measures.

**Figure 1:**
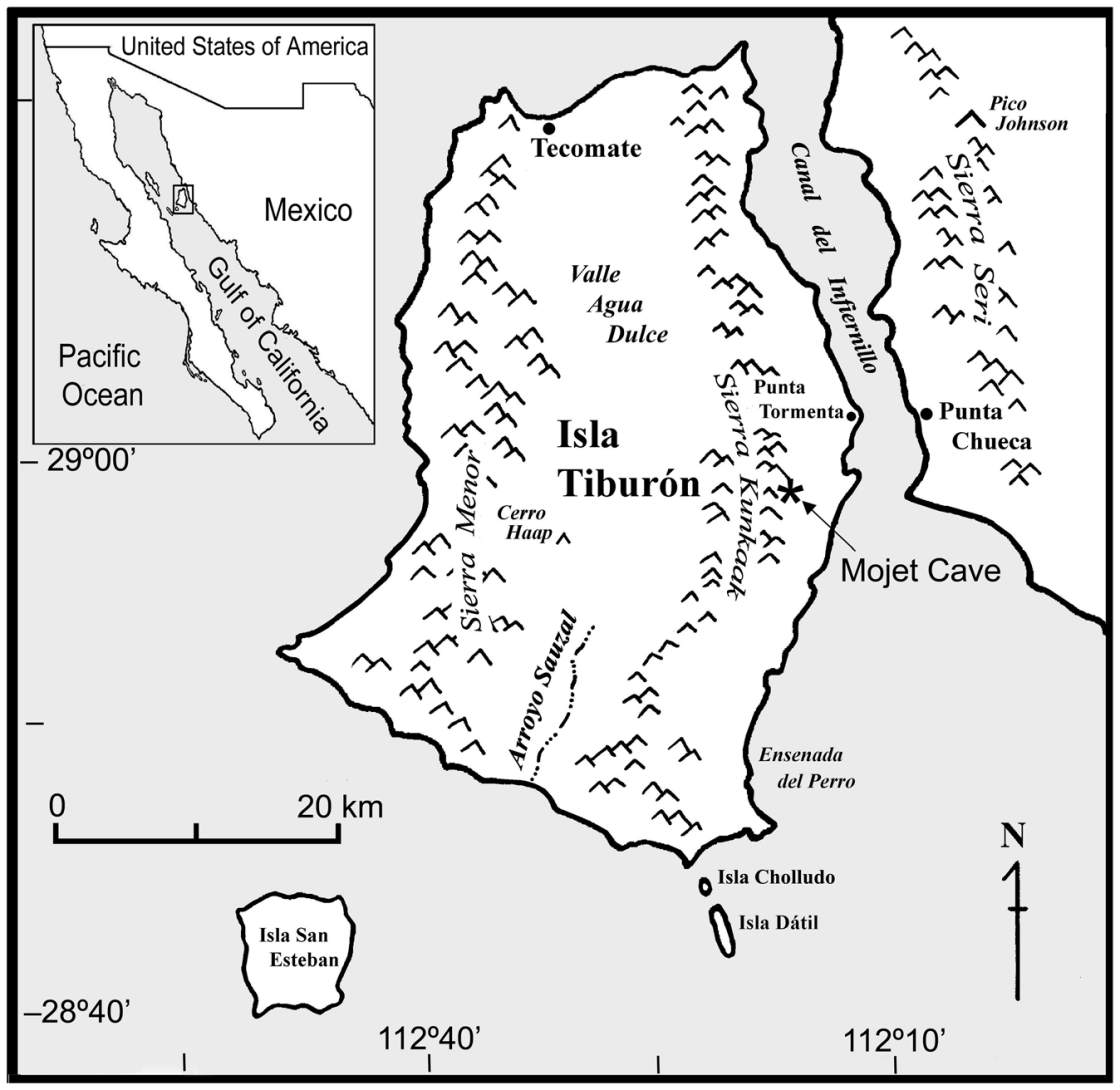
Tiburón Island in Gulf of California.

Here we report on the unintentional rewilding of bighorn sheep (*Ovis canadensis*) on Tiburón Island in the Gulf of California, a few kilometers off the west coast of the State of Sonora and the largest island in Mexico (1,218 km^2^; Fig. 1). The Canal del Infiernillo —a narrow (∼2–10 km wide) and shallow (∼5.5 m deep) channel —separates the island from the mainland and was submerged by rising sea level only 6,000–4,700 cal yrs B.P. [10]–[12].

Mountain ranges on the mainland (the Sierra Seri) and on the island (the Sierra Kunkaak) are very similar in area and suitable habitat. Mule deer (*Odocoileus hemionus*) and desert bighorn sheep (*Ovis canadensis nelsoni*, see end of methods for a discussion on bighorn sheep taxonomy) are found on the coastal mainland today, and pronghorn (*Antilocapra americana sonoriensis*) occurred along the coastal plains in the past [13]. Given that the island was part of the Sonoran mainland as recently as ca. 6,000 years ago, we expect the past occurrence of these species on the island. However, mule deer (*Odocoilus hemionus sheldoni*), an endemic subspecies, is the only non-introduced ungulate on Tiburón Island, and all previous accounts of bighorn sheep and pronghorn antelope correspond to modern attempts to introduce them [14].

The island has a diverse mammal fauna relative to other Gulf islands, but lacks top predators such as bobcat (*Lynx rufus*) and mountain lion (*Puma concolor*) that are common on the mainland. The reports of early explorers in the region confirm the historic absence of bighorn sheep on Tiburón. Charles Sheldon, an early 20^th^ century naturalist and avid sportsman, hunted bighorn sheep on the mainland ranges of coastal Sonora and mule deer on Tiburón Island in 1921, in each case accompanied by Seri guides. Sheldon's detailed field notes record the absence of bighorn sheep on Tiburón, as indicated in this passage: “The chief, Buro Alesan, tells me there are no wild sheep on Tiburón Island, but a few are in the Sierra Seri” [15], [16].

In 1975, sixteen female and four male desert bighorn sheep were introduced from the Sonoran mainland adjacent to Tiburón Island as a conservation measure [13], [17], [18]. The population grew rapidly to ∼500 animals, where it seems to have reached its carrying capacity [13], [19]–[21]. Low levels of hunting and other human disturbance, lack of mountain lion, absence of domesticated sheep and their contagious diseases, expansiveness of suitable habitat, and wetter conditions from 1976 to 1995 all probably contributed to introduction success. In agreement with early reports that bighorn sheep were not native to the island [15], all published studies about the transplanted population have treated the operation as an introduction of an alien species into a previously unoccupied ecosystem [13], [22].

For wildlife biologists, Tiburón Island has become both a long-term field experiment and an object lesson in conservation. The introduction of bighorn sheep was not only successful in establishing a viable population but, through translocations back to Sonora, Tiburón animals also have contributed significantly to recovery efforts on the mainland. In 1995, a coalition of institutions initiated an innovative program to fund bighorn sheep research and conservation while providing needed income for the Seri through international auctioning of exclusive hunting tags on the island. Starting in 1999, hunting permits garnered six-figure auction bids [23]; most recent prices range from US $80,000–90,000 a tag [24], [25]. Revenue from these auctions offers the Seri incentive to maintain Tiburón Island in an undisturbed state [13], [14], [26]. To date, this conservation story has been regarded as controversial due to the non-native status of bighorn sheep on the island. The impact of unchecked bighorn sheep herbivory on the island's Sonoran Desert flora, which includes several regional endemic species [26], was not considered prior to the introduction.

During a recent survey for fossil woodrat (*Neotoma*) middens on Tiburón Island, we discovered large pieces of an apparent sheep dung mat in Mojet Cave, a small rock shelter in the eastern foothills of the Sierra Kunkaak. Pellets from the recovered dung mat were ^14^C-dated to 1476–1632 calendar years before present (cal yr B.P.). We used morphological and ancient DNA (*a*DNA) analyses to determine the identity of the species that deposited the pellets. The ‘molecular caving’ [27] approach taken here adds a new dimension to the study of paleoenvironments in aridlands, and an opportunity to link theory and observations that address long-standing questions of lost populations and future conservation strategies.

## Results

### Morphological Identification

**Figure 2:**
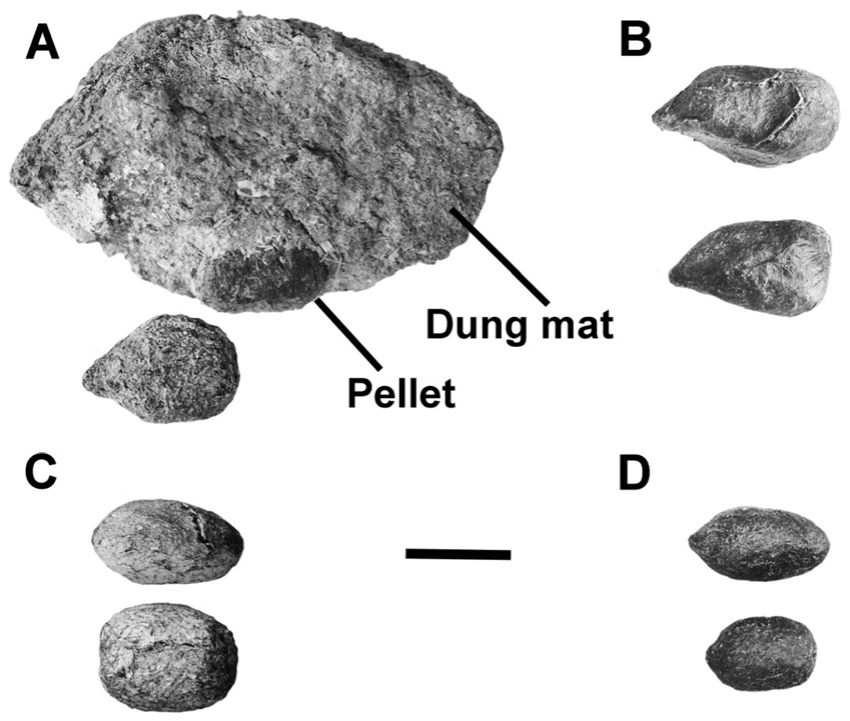
Dung from Mojet Cave and recent potential dung producers. A) dung pellet and mat (ETVP 4999) from Mojet Cave; identified as bighorn sheep, *Ovis canadensis* (see text). Note pellet in dung mat is featured below after removal. Opposite side of dung mat has a thin layer of bat guano adhering to the surface. Recent dung: B) bighorn sheep, *Ovis canadensis* (ETVP 6083); C) pronghorn antelope, *Antilocapra americana* (ETVP 6028); D) mule deer, *Odocoileus hemionus* (ETVP 6017). Scale bar equals 10 mm.

The Mojet Cave dung deposit contained both isolated complete pellets and those incorporated into an amorphous mat of crushed pellets; all were consolidated with crystallized urine. The pellets analyzed were small (averaging 15.5×10.1 mm; n = 3), showing the characteristically blunt proximal ends and pointed distal ends that attribute them to bighorn sheep (Fig. 2). This morphology indicated that the pellets represented a dry-season diet, typical of desert vegetation throughout most of the year. Desert bighorn sheep diets focus on diverse array of desertscrub species, especially succulents [26], [28]. Pellets of wapiti (*Cervus*; Cervidae), the extinct shrub-ox (*Euceratherium*; Ovibovinae), and the extinct Harrington's mountain goat (*Oreamnos harringtoni*; Rupicaprinae), all known to have inhabited mountains in the now arid Sonoran Desert during the late Pleistocene [29], are significantly larger in size, heavier, and have a conspicuously more robust pellet form [30], [31]. Pellets of extant deer and pronghorn (including the extinct *Stockoceros*, which is known to have frequented shallow rock shelters) are similar in size with those of *Ovis*, yet they are characteristically and typically longer in form (Fig. 2). Dung pellets of the living mountain goat (*Oreamnos americanus*) are distinctly smaller than those of *Ovis*. Thus, the morphology of the pellets from Mojet Cave suggests that bighorn sheep produced them.

### Ancient DNA

From exterior scrapings of several ancient pellets within dung mat sample B (see methods for sample descriptions), we sequenced one 90 base pair (bp) region of the 12S ribosomal RNA subunit of the mitochondrial genome (GenBank accession number KF769974) from DNA amplified in 12 replicate PCRs (7 forward, 5 reverse), and the three control region fragments from 2–4 replicate PCRs (78–117 bp each; KF769975 [3 forward, 4 reverse], KF769976 [3 forward, 3 reverse], KF769977 [3 forward, 2 reverse]). Neither the exterior scrapings of pellets from dung mat sample A nor the interior pellet material from either mat amplified successfully. Neither of the two extraction controls amplified at any locus, and none of the blank PCR controls amplified at the 12S or control region fragments. Despite the relatively short length of the target fragments (12S and control region), none of the PCR amplicons were successfully sequenced in their entirety in only one direction, but bidirectional reads could be aligned with each other. We saw no evidence of competing sequences (indicating contamination by multiple templates), and sequences were consistent over multiple PCR replicates, except for variation at one location in numbers of repeated base pairs (3 or 4) identified by different people reviewing the sequences.

**Figure 3:**
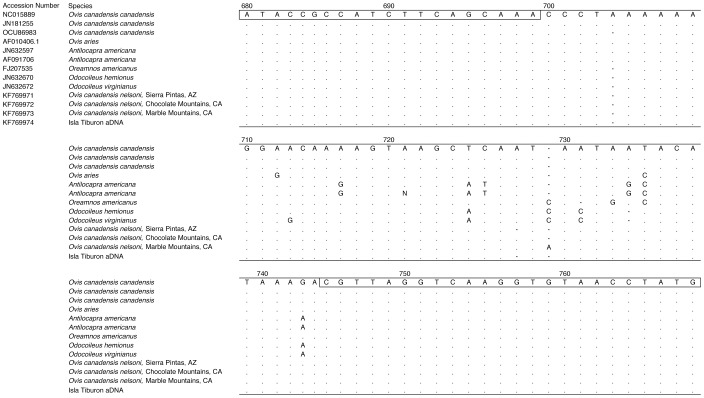
Isla Tiburón *a*DNA and Sonoran Desert ungulate sequences. Partial sequences of the 12S ribosomal RNA subunit of the mitochondrial genome for the Isla Tiburón *a*DNA sample and published sequences from all ungulate species thought to have existed in the area since the start of the Holocene, with GenBank accession numbers. Open box denotes primer region, dot indicates identical bases between sequences, N indicates unknown base. Sequence position numbers are derived from *O. c. canadensis* haplotype (GenBank Accession NC015889).

From a BLAST search, the consensus sequence for the 12S region of the midden sample clearly matched known bighorn sheep sequences, with 1–2 differences from published *O. c. canadensis* (Rocky Mountain bighorn sheep) sequences, and 0–1 differences from published *O. c. nelsoni* (desert bighorn sheep) sequences (Fig. 3). The ancient pellet 12S sequence differed from reference sequences for *Antilocapra americana* by 8 bp, from *Odocoileus hemionus* by 6 bp, from *Odocoileus virginianus* (white-tailed deer) by 7 bp, from *Oreamnos americanus* by 4 bp, from *Bos taurus* (domestic cattle) by 7 bp, and from *Ovis aries* (domestic sheep) by 3 bp (Fig. 3).

**Figure 4:**
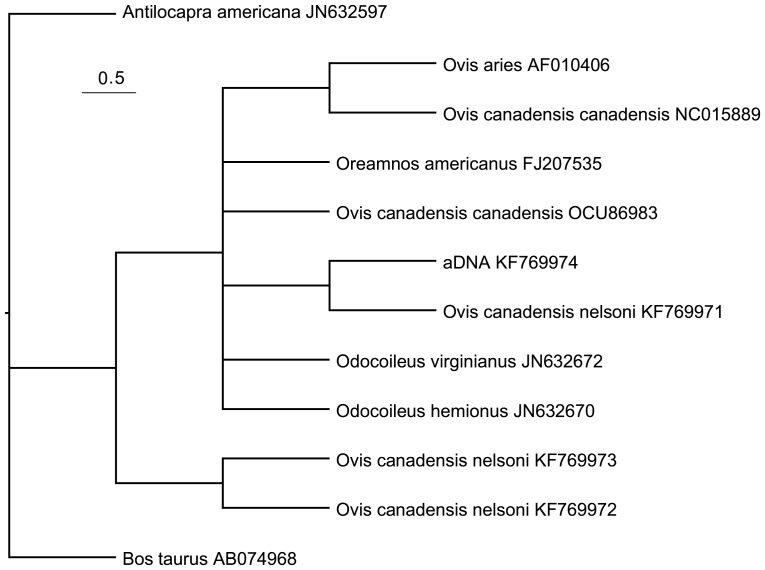
Phylogenetic tree for Isla Tiburón *a*DNA and ungulate sequences. Majority rule consensus tree from phylogenetic analysis of a 46ón *a*DNA sample and published sequences from all ungulate species thought to have existed in the area since the start of the Holocene, with GenBank accession numbers. The phylogeny was inferred using parsimony and treating indels as a 5^th^ character state.

Phylogenetic analysis of the 46 bp alignment of ancient and reference 12S sequences (after trimming primer sites) resulted in 9 equally parsimonious trees each with 24 steps, of which the majority rule consensus appears in Fig. 4. The ancient DNA sequence is identical to one of the modern bighorn samples, and clusters within a clade that includes all desert bighorn sheep samples as well as the Rocky Mountain bighorn, mountain goat, and domestic sheep individuals. The short length of the sequence alignment made it impossible to resolve relationships within this clade unambiguously, but the exact match of the ancient DNA to one of the modern desert bighorn sheep leaves no doubt about the identity of that sample.

In total, we sequenced 280 bp of three control region fragments from the ancient sample (Fig. S1). The ancient sequence exactly matched two published desert bighorn sheep haplotypes (*O. c. nelsoni* haplotypes B and V, GenBank accession numbers AY903995, AY904015). Haplotypes B and V were differentiated by only 4 sites outside the three fragments that we sequenced for this project, and originated from southern Mojave (B) [32] and the Sierra Pinta mountains of southern Arizona (V). The ancient sequence varied from each of the two known modern Tiburón bighorn sheep control region haplotypes (by 5 and 2 bp), and by one bp from a published sequence from Baja California identified as *O. c. weemsi* (Weem's desert bighorn sheep from Baja California). The ancient sequence varied from a published *O. c. canadensis* sequence originating in the Canadian Rocky Mountains [33] at 11 sites. Lastly, there were 53–57 differences between the ancient DNA sequence and published haplotypes for *Ovis aries* (Fig. 4).

## Discussion

The accumulation of plant and animal remains in aridland caves and rock shelters, sometimes imbedded in crystallized mammalian urine, offers unique opportunities for *a*DNA preservation and analysis. Although DNA molecules rapidly disintegrate after death and fossil DNA is highly fragmented [34], both the desiccation and protection within crystallized urine substantially slow DNA degradation [35]. Modern genetic techniques and diverse arrays of molecular primers for a multitude of taxa make it possible to analyze tiny amounts of DNA from dung, plant, and other material preserved in fossil middens [8]. In the case of Mojet Cave, the bighorn sheep pellets in the urine-hardened mat yielded low-quality DNA that could still be amplified via primers designed from extant material. However, extreme temperatures and temperature fluctuations, UV-radiation, and humidity in the area may have compromised the preservation of these samples, leading to fragmentation of DNA strands and the truncated sequences we observed.

We conducted extractions and PCR setup for ancient DNA samples in a laboratory with no history of mammalian genetic research, used new reagents, and observed standard *a*DNA practices [36]. We are confident that our *a*DNA sequences do not result from contamination. Sequence analysis of the *a*DNA recovered confirms that desert bighorn sheep deposited the pellets recovered from the Mojet Cave dung mat. Moreover, it is noteworthy that the *a*DNA control region haplotype does not match either of the two haplotypes recovered from 63 modern Tiburón sheep captured in 2007 [37] and previously reported in an unpublished study (GENBANK AY116622.1, AY116621.1).

We cannot specify whether bighorn sheep persisted or repeatedly colonized Tiburón Island during the Holocene, or pinpoint when or why they became locally extinct during the past 1500 years. Population model simulations under two future climate change scenarios show that extinction risk for the Tiburón bighorn sheep population increases more with mean drought severity than with drought variability [38]. According to the tree-ring record, mean drought severity and variability have varied substantially during the past two millennia. Gridded reconstructions of the July Palmer Drought Severity Index for the two grid points nearest Tiburón [39] (centered at 27.5N, 110W and 30N, 112.6W) show increased mean drought severity from AD 400 to 1400, encompassing the Medieval Climate Anomaly (MCA). The tree-ring record is not long enough to evaluate if this dry period was unprecedented in the Holocene, causing a unique extinction on Tiburón. Oceanic conditions can induce periods of extreme drought in the coastal deserts of the Gulf of California [40], which can greatly increase risk of population extinction of bighorn sheep in desert environments [41], [42].

Bighorn sheep have a highly fragmented distribution through much of their current range, resembling a metapopulation in many regions, including the deserts of North America [43]. High rates of local population extinction in desert environments [41] and strong genetic drift [42] are counterbalanced to varying degrees by gene flow and recolonization, depending on population isolation and local climatic conditions [32], [42], [44]. In the absence of gene flow, the small population size characteristic of desert bighorn sheep populations results in very rapid genetic drift and likely inbreeding [44]. This situation is further exacerbated by the stochastic nature of precipitation and forage availability in Sonoran Desert systems, and potential hunting by indigenous peoples. The Seri lived off the seasonal bounty of the desert and sea [16], [45], and it can be safely assumed that they occasionally hunted bighorn sheep. Bighorn sheep remains figure prominently in the archeological record of the Southwest, ranging from cremated remains in the Pinacate Mountains in northwestern Mexico [46] to widespread evidence of bighorn as a food resource in southern Arizona to the Colorado Plateau [47], [48].

We hypothesize that isolation of the prehistoric Tiburón bighorn sheep population resulting from sea level rise, combined with subsequent drivers that act on small populations, including inbreeding, overharvesting by hunters, and megadroughts typical of Northern Mexico and the Southwestern U.S.A., figured in their local extinction. For similar reasons, the re-introduced population on Tiburón Island likewise is vulnerable to extinction. Genetic diversity of the island population is demonstrably low, attributed mainly to genetic drift and lack of gene flow with other populations [37], [49]; low genetic diversity has been correlated with lower fitness in bighorn sheep [50]. The Tiburón population also was modeled to be susceptible to stochastic effects via increased aridity and warmer temperatures associated with climate variability and change [51], as well as and continued extraction of animals for repopulation efforts on the mainland [38].

Confirmation of the prior presence and local extinction of bighorn sheep on Tiburón Island refutes their status as a non-native species. This extended baseline anchors bighorn sheep within the changing ecology of Tiburón Island, furthers their importance as a focal point for conservation and management, and presents a cautionary tale. The introduction of bighorn sheep on Tiburón in 1975 is more justifiable than realized at the time. It raises other questions, however. Now that we know that bighorn sheep were native to the island, does this necessarily quell concern about the impacts of reintroduction? Did mountain lion, their main predator on the mainland, also occur on the island but suffer extinction at the same time as bighorn sheep? Does the absence of predators matter, or is it mimicked by current levels of trophy hunting that focuses on the healthiest animals, and not on the weak?

As evidenced by local extinction on Tiburón and throughout their range [44], bighorn sheep, as other large ungulate species, face increased extinction rates from future droughts exacerbated by warming [38], [52]. The Tiburón bighorn sheep population is of critical significance as a source population for mainland introductions, great economic and cultural importance for the Seri community, and an indicator of environmental conditions. Careful management of trophy hunting and introductions of new animals from the mainland to broaden genetic diversity could help avoid another local extinction.

So how should we regard the reintroduction of bighorn sheep now that we know how recently it occurred naturally on the island? Is it a restoration or a biological invasion? This question should apply to most cases of rewilding and de-extinction efforts. Native plant communities on Tiburón Island clearly co-evolved with bighorn sheep and, given the short time since local extinction, should still be resilient to the perturbations caused by renewed herbivory. It is unlikely that local plants lost their defenses since the sheep's extinction, which happened sometime between 1500 and 100 years ago. Mountain lion may or may not have also occurred on the island, and predator pressure on the introduced population now may be greatly diminished.

In other cases of rewilding, exactly how long is required for the remaining native species to evolve and lose their defenses and resilience? Native horses have been gone from North America for more than 10,000 years, and so have most of their predators. This could have had evolutionary consequences in plant and animal communities that eroded their potential resilience to rewilding. Even if bighorn “belong” on Tiburón, as well as horses in North America, does this necessarily absolve wildlife managers and conservationists from considering any and all unintended impacts of rewilding?

Finally, we recognize that our discovery of the bighorn sheep deposit in a remote cave on Tiburon Island also was unintentional. We did not set out to test whether or not bighorn sheep were native to the island; we were actually looking for something else. We now envision strategic and purposeful ‘molecular caving’ to pinpoint the timing and circumstances for other Holocene extinctions, and thus inform conservation efforts, in the Gulf of California and other arid regions worldwide.

## Methods

To better understand past plant communities and the origin of the modern Sonoran Desert, fossil packrat (*Neotoma* spp.) middens were collected on Tiburón Island in March 2012. Among the middens we found a different type of urine-hardened deposit containing ungulate dung in a rock shelter at 235 m elevation on Hast Coopol, a low-lying volcanic peak on the eastern foothills of the Sierra Kunkaak (Fig. 1).

Thick crusts of urine-hardened sheep dung commonly line the floors of caves and rock shelters occupied by both wild and domestic sheep worldwide. Domestic sheep (*Ovis aries*) are not known to have inhabited the island. Bighorn sheep (*O. canadensis*) are the only late Pleistocene wild sheep (*Ovis* sp.) known to inhabit the Intermountain West and south into Mexico [29], [53], [54]. Unlike other extant Sonoran Desert artiodactyls (e.g., pronghorn, and mule deer), bighorn sheep commonly use caves and rock overhangs to bed and escape the midday heat in summer [55] and their kidneys concentrate urine to conserve water [56]. As it evaporates, the viscous urine can crystallize and cement both sediment and dung on the cave floor, much like the process that forms packrat and other rodent middens [57].

The rock shelter on Tiburón Island named here Mojet Cave, (mojet ['moxεt], the Seri name for bighorn sheep) [58] is about 8 m deep and 15 m wide (Video S1). Two blocks or mats of artiodactyl dung (sample A consisted of two small pieces both ∼20×10×8 cm, and sample B one large mat ∼40×30×10 cm) were collected amid roof fall on the cave floor, following the same protocols used for sampling packrat middens [59]. Five fecal pellets taken from both the top and bottom layers of dung mat sample B were sent to the UC, Irvine W. M. Keck Carbon Cycle Accelerator Mass Spectrometry Laboratory for radiocarbon dating. All pellets dated fell within the age range of 1595–1725±20 ^14^C yr B.P., which was calibrated to a mean age of 1476–1632 calendar years before present (cal yr B.P.) using the Intcal13 calibration [60]. A pellet from the bottom of the midden dated to 1530±15 ^14^C yr B.P. and one from the top as 1625±20 ^14^C yr B.P. That the dates are stratigraphically reversed indicates the deposit likely formed at once. Two independent methods, morphological identification and ancient DNA analysis were used to identify the fossil pellets. The same pellets used for *a*DNA extraction were dated to 1720±20 and 1725±20 ^14^C yr B.P. Those used for morphological identification were not dated to preserve the intact nature of the pellet and are deposited in the East Tennessee Vertebrate Paleontology Laboratory (Eastern Tennessee State University) under collection number ETVP 4999. All necessary permits were obtained for the described study, which complied with all relevant regulations. The material for this study was collected under collector's permit FAUT–0265 granted to Exequiel Ezcurra by the Secretaría de Medio Ambiente y Recursos Naturales (SEMARNAT; authorization document SGPA/DGVS/02213-13).

### Morphological Identification

We compared identically sized and shaped pellets as the one dated with an extensive dung collection of both modern and extinct herbivores housed in the East Tennessee Vertebrate Paleontology Laboratory. Isolate dung pellets of adult *Cervus* and the extinct *Euceratherium*, *Symbos*, and *Oreamnos harringtoni* typically have a ratio of the width∶length (measurements in mm) versus the weight (g) distinctly greater than that produced by *Ovis*, *Antilocapra*, and the living *Oreamnos americanus*; weights greater than 0.5 g readily distinguish these larger ungulates from *Ovis* and *Antilocapra* [31]. Isolated pellets of living adult *Ovis* spp., *Oreamnos*, *Antilocapra*, and the extinct *Stockoceros* can overlap in weight (all typically weigh less than 0.3 g in weight). When considering size/shape, pronghorns will more often have a longer pellet, with width∶length ratios greater than 1.1, while most often *Ovis* spp. produce a pellet ratio between 0.9 to 0.5, creating their more cuboid appearance [30]. This can vary with a more boreal and green vegetation diet. The classification of the pellets from Mojet Cave as *Ovis* is based on gross morphology and should be viewed as a ‘best fit’ identification.

### Ancient DNA

To substantiate our identification from pellet morphology we also sequenced DNA from the fossilized pellets. We observed basic tenets of ancient DNA handling [36], [61], [62] by 1) restricting all handling of material prior to PCR amplification to a laboratory at Oregon State University that had never been used for genetic research or mammalian research of any kind; 2) restricting laboratory equipment used in all pre-PCR operations to equipment that had never been used with mammalian genetic samples; 3) only allowing personnel to enter the ancient DNA laboratory if they had not previously entered the modern DNA laboratory that day. The modern DNA laboratory is on a different floor within the same building. We used only newly-purchased reagents, bleached surfaces between extractions, and autoclaved supplies. We attempted to extract and amplify DNA from pellets from two different dung deposits within the larger dung mat (sample B). We scraped the exterior of pellets (where epithelial cells are concentrated, [63]) with a bleached and flamed razor blade, but also collected dust from the interior of the pellets for a second extraction, in the event that the exterior surface had been contaminated or degraded. Due to the small size of the pellets, we used two pellets for each extraction of surface material; inner material for the second extraction was taken from a single pellet. We used the Aquagenomics/AquaPrecipi DNA extraction kit (Multitarget Pharmaceuticals, Utah), to extract DNA from 0.03 g of scraped dust from each replicate. We added 15 mAU of proteinase K (Qiagen, California) and approximately 0.2 mL of 1.0 mm zirconia beads (Biospec, Oklahoma) to the dust, vortexed briefly to lyse the cells, and incubated the samples for one hour at 60°C. Using the same protocol, we conducted two blank extractions to detect contamination of reagents or supplies. Samples were rehydrated with 100 uL of 1× TE buffer (pH 8.0) and stored at 4°C.

We attempted to amplify fragments of varying lengths to evaluate the *a*DNA principle that amplification success will increase with decreasing fragment size [36]. As expected, efforts to amplify a 350 bp fragment of the 16S ribosomal RNA gene (Table S1) that has previously been used for species identification of ungulate pellets were unsuccessful, with the exception of one contaminated PCR negative that was shown by BLAST-search to have amplified human DNA. Instead, we used Primer3 [64], [65] to design primer pairs to amplify a ∼90 bp fragment of the 12S ribosomal RNA gene on the mitochondrial genome (Table S1). Using sequences published on GenBank (see Table S2 for accession numbers), we designed primers for locations that we identified as largely conserved but with variation between priming sites across mule and white tailed deer (*Odocoileus hemionus* and *Odocoileus virginianus*, respectively), mountain goats (*Oreamnos americanus*), pronghorn antelope (*Antilocapra americana*), domestic sheep (*Ovis aries*), domestic cattle (*Bos taurus*), and bighorn sheep (*Ovis canadensis*). We used the polymerase chain reaction (PCR) to amplify this gene fragment in reactions consisting of 5× Qiagen Multiplex PCR master mix, 5 µg bovine serum albumin, 0.2 µM of each primer, and 0.5 µL of DNA extract and brought the reaction to a 10 µL reaction volume with nuclease-free molecular grade water. Thermalcycling conditions were as follows: 15 minutes at 95°C, followed by 35 cycles of [95°C for 30 s, 54°C for 90 s, 72°C for 60 s] and a final elongation of 30 minutes at 60°C. We included a negative control (molecular grade water) in each PCR run to monitor for contamination, and also attempted to amplify the two negative extraction controls. We verified amplification using an agarose gel stained with GelRed (Biotium, California) and used 2 µL shrimp alkaline phosphatase (ExoSAP-IT, Affymetrix, Santa Clara, CA) to prepare 4 µL of DNA from each amplicon for sequencing. PCR products were submitted to the Molecular Research Core Facility at Idaho State University for bidirectional sequencing on an ABI 3130XL DNA analyzer. We also sequenced the same section of the12S gene in one bighorn sheep sample from each of: Chocolate Mountains (CA), Marble Mountains (CA), Orocopia Mountains (CA), and the Sierra Pintas (AZ). These samples were chosen to assess potential variation in the 12S gene in desert bighorn sheep across a broad geographical area, and because this subspecies (*O. c. nelsoni*) was not represented in GenBank at this 12S gene.

To compare variation between the fossil haplotypes and those of modern bighorn sheep we also designed primers for three short sections of the mitochondrial DNA control region (∼80–120 bp each; Table S1; Fig. S1) within a 515 bp region previously sequenced for numerous modern bighorn sheep populations [33]. Because of the highly degraded nature of ancient DNA, we did not attempt to sequence this entire region, but chose three sections that 1) showed variability in published sequences, and 2) supported reliable primers in conserved regions. We designed primers using sequences published on GenBank for bighorn sheep described as *O. c. nelsoni*, *O. c. weemsi*, and *O. c. mexicana* to capture variation across a wide range of desert bighorn sheep (Table S2), and included two published haplotypes from bighorn sheep descended from individuals translocated to Isla Tiburón during the 1970s (accession numbers AY116621 and AY116622, Table S2). Amplification of the control region fragments followed the same PCR recipe and cycling conditions as 12S.

Because of the degraded nature of most ancient DNA and the possibility that base modifications over time could alter the sequences, for the ancient pellets, we attempted to amplify and sequence each targeted region in a minimum of three separate PCR reactions. Raw chromatogram traces were trimmed to the length at which all bases could be unambiguously identified. We verified base calls, searched each sequence on NCBI BLAST, and aligned all replicate sequences in Geneious (v.6.1.2; Biomatters, available from http://www.geneious.com/) and had two other experienced researchers independently do the same. In case of a discrepancy between calls, we reviewed sequences again with a fourth experienced observer and corrected obvious errors, after which we selected the call made by the majority of observers. Discrepancies, when they arose, typically resulted from difficulty in interpreting strings of single repeated base pairs (e.g., AAAA). We generated consensus sequences, resolving discrepancies based on majority of calls and re-evaluation of the original sequences, and compared them with sequences published on Genbank for other candidate species (Table S2). In that comparison, we included 4 published sequences from domestic sheep (*O. aries*, GenBank AF010406, EF490455, NC001941, and HE577848) and two from domestic cattle (*Bos taurus*, GenBank AB074968 and AF492351) to rule out contamination from domestic animals in our lab reagents. Published sequences were selected from different studies to capture intraspecific diversity. However, as we observed no intraspecific diversity in these two species for this 12S fragment, we included only one example for each species in further analyses.

To confirm the species identity of the ancient DNA sample and rule out the possibility of contamination from other mammals, we conducted a phylogenetic analysis on the ancient 12S sequence and reference sequences for other wild and domestic artiodactyls that we downloaded from GenBank (a 46 bp region after trimming primers). While molecular data are most frequently analyzed with model-based likelihood or Bayesian methods, those methods treat alignment gaps due to insertions and deletions as missing data, and require moderately long sequence alignments in order to infer an appropriate model of nucleotide substitution. Our very short ribosomal alignment includes several indels that clearly demonstrate phylogenetic signal; treating these as missing data in a pilot likelihood analysis resulted in a loss of almost all phylogenetic resolution in the resulting consensus. To capture the signal in the indels, we inferred a phylogeny under parsimony in PAUP* 4.0 beta [66], while treating gaps as a fifth character state, using *Antilocapra americana* as the outgroup and conducting 1000 heuristic searches with random addition sequences.

The taxonomy of bighorn sheep subspecies remains somewhat confused. Cowan originally described seven subspecies of bighorn sheep [67]. However, recent genetic and morphometric evaluations [68]–[73] demonstrated that there are likely only three valid subspecies: *O. c. canadensis* (including bighorn sheep formerly described as *O. c. canadensis*, *O. c. auduboni* and *O. c. californiana* excepting populations in the Sierra Nevada mountains of California), *O. c. nelsoni* (desert bighorn sheep, including bighorn sheep formerly described as *O. c. nelsoni*, *O. c. mexicana*, *O. c. cremnobates*, and likely *O. c. weemsi* although insufficient specimens were available to confirm the validity of *O. c. weemsi*), and *O. c. sierrae* (populations in the Sierra Nevada Mountains of California, previously described as *O. c. californiana*).

## Supporting Information

Figure S1 **Control regions of Isla Tiburón** ***a*** **DNA and select bighorn sheep populations.** Partial control region sequences of Isla Tiburón *a*DNA sample and published bighorn sheep samples, sequenced as three fragments (a–c). Open boxes denote primer region, dot indicates identical bases between sequences, dash indicates deletion, N indicates unknown base. Sequence position numbers are derived from the reverse complement of *O. c. nelsoni* haplotype B [44] (GenBank Accession AY903995).
(PDF)

Table S1 **Primer sequences and fragment size.** The genes sequenced or attempted from ancient fecal pellets from Tiburón Island, Mexico.
(PDF)

Table S2 **Accession numbers of sequences used.** The sequences used for primer development and sequence alignment for reference sequences and ancient fecal DNA of unknown origin from Tiburón Island, Mexico.
(PDF)

Video S1 **Movie of Mojet Cave, Tiburón Island.** The video shows the fossil dung mat as encountered before any sampling or manipulation was made. Taken by Wilder 29 Mar 2012, featured in the video (in order of appearance) Andrew Semotiuk, Carlos Armando Mendes Romero, Jose Ramon Torres Molina.
(MP4)
