# Supplementary material for: Local Extinction and Unintentional Rewilding of Bighorn Sheep (Ovis canadensis) on a Desert Island
Source: PLoS One. 2014 Mar 19;9(3):e91358. doi: 10.1371/journal.pone.0091358 (PMC3960132; doi:10.1371/journal.pone.0091358)
Supplement: Figure S1 — Control regions of Isla Tiburón a DNA and select bighorn sheep populations. Partial control region sequences of Isla Tiburón aDNA sample and published bighorn sheep samples, sequenced as three fragments (a–c). Open boxes denote primer region, dot indicates identical bases between sequences, dash indicates deletion, N indicates unknown base. Sequence position numbers are derived from the reverse complement of O. c. nelsoni haplotype B [44] (GenBank Accession AY903995). (PDF) [file pone.0091358.s001.pdf]

a)

Accession Number	Species	Positions 6–40	Positions 41–75	Positions 76–83
		10, 20, 30, 40	50, 60, 70	80
AY903995	*O. c. nelsoni,* haplotype B	AGCGGGTTGTTGGTTTCACGCGGCATGGTAGATAA	GCTCGTGATCTAGTGGACAGGATACGCATGTTGAC	TAGAACGG
AY904015	*O. c. nelsoni,* haplotype V	....	....	....
AY903996	*O. c. nelsoni,* haplotype C	....	....	....
AY904011	*O. c. nelsoni,* haplotype R	....	....	....
AY904013	*O. c. nelsoni,* haplotype T	.............................G.....	..................G................	....
AY116623	*O. c. weemsi*	....	....	....
AY116621	*O. c. mexicana,* Tiburon-haplotype1	................................C..	....	....
AY116622	*O. c. mexicana,* Tiburon-haplotype2	....	....	....
AF010407	*Ovis aries*	.............................GA.C..	..................G.....T..........	...G....
AY091499	*Ovis aries*	.............................GA.C..	............A......................	...A.T..
KF769975	Isla Tiburon aDNA	...............N...................	.NNNNNN............................	....
AY091486	*O. c. canadensis*	....	.....C..........G..................	.G.G....

b)

Accession Number	Species						70										80										90									
AY903995	*O. c. nelsoni,* haplotype B	C	G	C	A	T	G	T	T	G	A	C	T	A	G	A	A	C	G	G	A	T	T	T	G	A	C	T	T	T	A	T	G	T	G	C
AY904015	*O. c. nelsoni,* haplotype V	.	.	.	.	.	.	.	.	.	.	.	.	.	.	.	.	.	.	.	.	.	.	.	.	.	.	.	.	.	.	.	.	.	.	.
AY903996	*O. c. nelsoni,* haplotype C	.	.	.	.	.	.	.	.	.	.	.	.	.	.	.	.	.	.	.	.	.	.	.	.	.	.	.	.	.	.	.	.	.	.	.
AY904011	*O. c. nelsoni,* haplotype R	.	.	.	.	.	.	.	.	.	.	.	.	.	.	.	.	.	.	.	.	.	.	.	.	.	.	.	.	.	.	.	.	.	.	.
AY904013	*O. c. nelsoni,* haplotype T	.	.	.	.	.	.	.	.	.	.	.	.	.	.	.	.	.	.	.	.	.	.	.	.	.	.	.	.	.	.	.	.	.	.	.
AY116623	*O. c. weemsi*	.	.	.	.	.	.	.	.	.	.	.	.	.	.	.	.	.	.	.	.	.	.	.	.	.	.	.	.	.	.	.	.	.	.	.
AY116621	*O. c. mexicana,* Tiburon-haplotype1	.	.	.	.	.	.	.	.	.	.	.	.	.	.	.	.	.	.	.	.	.	.	.	.	.	.	.	.	.	.	.	.	.	.	.
AY116622	*O. c. mexicana,* Tiburon-haplotype2	.	.	.	.	.	.	.	.	.	.	.	.	.	.	.	.	.	.	.	.	.	.	.	.	.	.	.	.	.	.	.	.	.	.	.
AF010407	*O. aries*	T	.	.	.	.	.	.	.	.	.	.	.	.	.	G	.	.	.	.	.	.	.	.	.	.	.	.	.	C	.	.	.	.	A	.
AY091499	*O. aries*	.	.	.	.	.	.	.	.	.	.	.	.	.	.	A	.	T	.	.	.	.	.	.	.	.	.	.	.	C	.	.	.	.	A	.
KF769976	Isla Tiburon aDNA	.	.	.	.	.	.	.	.	.	.	.	.	.	.	.	.	.	.	.	.	.	.	.	.	.	.	.	.	.	.	.	.	.	.	.
AY091486	*O. c. canadensis*	.	.	.	.	.	.	.	.	.	.	.	.	G	.	G	.	.	.	.	.	.	.	.	.	.	.	.	.	.	.	.	.	.	.	.

	100										110										120										130				
O. c. nelsoni, haplotype B	C	A	T	G	T	A	C	G	A	A	C	A	A	G	C	A	G	T	T	G	A	A	T	G	T	C	C	C	A	T	G	T	A	C	T
O. c. nelsoni, haplotype V	.	.	.	.	.	.	.	.	.	.	.	.	.	.	.	.	.	.	.	.	.	.	.	.	.	.	.	.	.	.	.	.	.	.	.
O. c. nelsoni, haplotype C	.	.	.	.	.	.	.	.	.	.	.	.	.	.	.	.	.	.	.	.	.	.	.	.	.	.	.	.	.	.	.	.	.	.	.
O. c. nelsoni, haplotype R	.	.	.	.	.	.	.	.	.	.	.	.	.	.	.	.	.	.	.	.	.	.	.	.	.	.	.	.	.	.	.	.	.	.	.
O. c. nelsoni, haplotype T	.	.	.	.	.	.	.	.	.	.	.	.	.	.	.	.	A	.	.	.	.	.	.	.	.	.	.	.	.	.	.	.	.	.	.
O. c. weemsi	.	.	.	.	.	.	.	.	.	.	.	.	.	.	.	.	.	.	.	.	.	.	.	.	.	.	.	.	.	.	.	.	.	.	.
O. c. mexicana, Tiburon-haplotype1	.	.	.	.	.	.	.	.	.	.	.	.	.	.	.	.	.	.	C	.	.	.	.	.	.	.	.	.	.	.	.	.	.	.	.
O. c. mexicana, Tiburon-haplotype2	T	.	.	.	.	.	.	.	.	.	.	.	.	.	.	.	.	.	.	.	.	.	.	.	.	.	.	.	.	.	.	.	.	.	.
O. aries	T	.	.	.	.	.	.	.	G	T	.	.	.	.	.	.	.	.	.	C	.	.	.	.	C	.	.	T	.	C	A	.	G	.	.
O. aries	T	.	.	.	.	.	.	.	G	T	.	.	.	.	.	.	.	.	.	T	.	.	.	A	C	.	.	T	.	C	A	.	G	.	.
Isla Tiburon aDNA	.	.	.	.	.	.	.	.	.	.	.	.	.	.	.	.	.	.	.	.	N	.	.	.	.	.	.	.	.	.	.	.	.	.	.
O. c. canadensis	.	.	.	.	.	.	.	.	.	.	.	G	.	.	.	.	A	.	.	.	.	.	.	.	.	.	.	.	.	.	.	.	.	.	.

						140										150										160								
O. c. nelsoni, haplotype B	T	T	A	A	T	G	A	A	G	T	T	A	A	T	G	T	G	C	T	T	G	C	T	T	A	T	A	T	G	C	A	T	G	G
O. c. nelsoni, haplotype V	.	.	.	.	.	.	.	.	.	.	.	.	.	.	.	.	.	.	.	.	.	.	.	.	.	.	.	.	.	.	.	.	.	.
O. c. nelsoni, haplotype C	.	.	G	.	.	.	.	.	.	.	.	.	.	.	.	.	.	.	.	.	.	.	.	.	.	.	.	.	.	.	.	.	.	.
O. c. nelsoni, haplotype R	.	.	G	G	.	.	.	.	.	.	.	.	.	.	.	.	.	.	.	.	.	.	.	.	.	.	.	.	.	.	.	.	.	.
O. c. nelsoni, haplotype T	.	.	.	G	.	.	.	.	A	.	.	.	.	.	.	.	.	.	.	.	.	.	.	.	.	.	.	.	.	.	.	.	.	.
O. c. weemsi	.	.	.	.	.	.	.	.	.	.	.	.	.	.	.	.	.	.	.	.	.	.	.	.	.	.	.	.	.	.	.	.	.	.
O. c. mexicana, Tiburon-haplotype1	.	.	G	G	.	.	.	.	.	.	.	.	.	.	.	.	.	.	.	.	.	.	.	.	.	.	.	.	.	.	.	.	.	.
O. c. mexicana, Tiburon-haplotype2	.	.	.	.	.	.	.	.	.	.	.	.	.	.	.	.	.	.	.	.	.	.	.	.	.	.	.	.	.	.	.	.	.	.
O. aries	.	C	.	G	.	.	.	.	.	.	G	.	.	.	.	.	A	.	A	.	.	.	C	.	.	.	.	C	.	.	.	.	.	.
O. aries	.	C	.	G	.	.	.	.	A	C	A	.	.	.	.	.	A	.	A	.	.	.	.	.	.	.	.	.	.	.	.	.	.	.
Isla Tiburon aDNA	.	.	.	.	.	.	.	.	.	.	.	.	.	.	.	.	.	.	.	.	.	.	.	.	.	.	.	.	.	.	.	.	.	.
O. c. canadensis	.	.	.	.	.	.	.	.	.	.	C	.	.	.	.	.	A	.	.	.	.	.	.	.	.	.	.	.	.	.	.	.	.	A

c)

```
Accession Number  Species                                  320                 330                 340
AY903995  O. c. nelsoni, haplotype B             A C A T T C A A G G T G G A C A T A G G G T A T A T T - G A T T T G T G
AY904015  O. c. nelsoni, haplotype V             . . . . . . . . . . . . . . . . . . . . . . . . . . . . . . . . . . . .
AY903996  O. c. nelsoni, haplotype C             . . . . . . . . . . . . . . . . . . . . . . . C . . . . . . . . . . . .
AY904011  O. c. nelsoni, haplotype R             . . . . . . . . . . . . . . . . . . . . . . . C . . . . . . . . . . . .
AY904013  O. c. nelsoni, haplotype T             . . . . . . . . . . . . . . . . . . . . . . . C . . . . . . . . . . . .
AY116623  O. c. weemsi                           . . . . . . . . . . . . . . . . . . . . . . . C . . . . . . . . . . . .
AY116621  O. c. mexicana, Tiburon-haplotype1     . . . . . . . . . . . . . . . . . . . . . . . C . . . . . . . . . . . .
AY116622  O. c. mexicana, Tiburon-haplotype2     . . . . . . . . . . . . . . . . . . . . . . . . . . . . . . . . . . . .
AF010407  O. aries                               . . . . . . . . . A C A . . . . . . A . A . . . . . . G . G . . . A . .
AY091499  O. aries                               . T G . . T . . . A C A . . . . . . A . A . . . G . . G . G . . . A . .
KF769977  Isla Tiburon aDNA                      . . . . . . . . . . . . . . . . . . . . . . . . . . . . . . . . . . . .
AY091486  O. c. canadensis                       . . . . . . . . . . . . . . . . . . . . . C . C . . . . . . . . . . . .

                                                                     350                 360                 370
          O. c. nelsoni, haplotype B             - - - - - - G A T G T C C G T G G G T G G A T G G - T G T G C T T T T C
          O. c. nelsoni, haplotype V             . . . . . . . . . . . . . . . . . . . . . . . . . . . . . . . . . . . .
          O. c. nelsoni, haplotype C             . . . . . . . . . . . . . . . . . . . . . . . . . . . . . . . . . . . .
          O. c. nelsoni, haplotype R             . . . . . . . . . . . . . . . . . . . . . . . . . . . . . . . . . . . .
          O. c. nelsoni, haplotype T             . . . . . . . . . . . . . . . . . . . . . . . . . . . . . . . . . . . .
          O. c. weemsi                           . . . . . . . . . . . . . . . . . . . . . . . . . . . . . . . . . . . .
          O. c. mexicana, Tiburon-haplotype1     . . . . . . . . . . . . . . . . . . . . . . . . . . . . . . . . . . . .
          O. c. mexicana, Tiburon-haplotype2     . . . . . . . . . . . . . . . . . . . . . G . . . . . . . . . . . . . .
          O. aries                               A A C G C T C G . . . . . . . . . . . . . . . . . T . . . . . . . . . .
          O. aries                               A A C G C T C . . . . . . . . . . . . . . G . . . T . . . . . . . . . .
          Isla Tiburon aDNA                      . . . . . . . . . . . . . . . . . . . . . . . . . . . . . . . . . . . .
          O. c. canadensis                       . . . . . . . . . . . . . . . . . . . . . . . . . . . . . . . . . . . .

                                                           380                 390                 400                 410
          O. c. nelsoni, haplotype B             T T G T A T G G G T A T G G G T T G T T G T T A T G T G A G C T T G T G
          O. c. nelsoni, haplotype V             . . . . . . . . . . . . . . . . . . . . . . . . . . . . . . . . . . . .
          O. c. nelsoni, haplotype C             . . . . . . . . . . . . . . . . . . . . . . . . . . . . . . . . . . . .
          O. c. nelsoni, haplotype R             . . . . . . . . . . . . . . . . . . . . . . . . . . . . . . . . . . . .
          O. c. nelsoni, haplotype T             . . . . . . . . . . . . . . . . . . . . . . . . . . . . . . . . . . . .
          O. c. weemsi                           . . . . . . . . . . . . . . . . . . . . . . . . . . . . . . . . . . . .
          O. c. mexicana, Tiburon-haplotype1     . . . . . . . . . . . . . . . . . . . . . . . . . . . . . . . . . . . .
          O. c. mexicana, Tiburon-haplotype2     . . . . . . . . . . . . . . . . . . . . . . . . . . . . . . . . . . . .
          O. aries                               . . . . . . . . . - - - - - - - - - . . . . . . . . . . G . T . . . . .
          O. aries                               . . . . . . . . . - - - - - - - - - . . . . . . . . . . G . . . . . . .
          Isla Tiburon aDNA                      . . . . . . . . . . . . . . . . . . . . . . . . . . . . . . . . . . . .
          O. c. canadensis                       . . . . . . . . . . G . . . . . . . . . . . . . . . . . . . . . . . . .

                                                                   420
          O. c. nelsoni, haplotype B             G A G T G G A A A G T C C G T G T
          O. c. nelsoni, haplotype V             . . . . . . . . . . . . . . . . .
          O. c. nelsoni, haplotype C             . . . . . . . . . . . . . . . . .
          O. c. nelsoni, haplotype R             . . . . . . . . . . . . . . . . .
          O. c. nelsoni, haplotype T             . . . . . . . . . . . . . . . . .
          O. c. weemsi                           . . . . . . . . . . . . . . . . .
          O. c. mexicana, Tiburon-haplotype1     . . . . . . . . . . . . . . . . .
          O. c. mexicana, Tiburon-haplotype2     . . . . . . . . . . . . . . . . .
          O. aries                               . . . . . . G . . . . . . . . . .
          O. aries                               . . . . . . G . . . . . . . . . .
          Isla Tiburon aDNA                      . . . . . . . . . . . . . . . . .
          O. c. canadensis                       . . . . . . . . . . . . . . . . .
```
