# Supplementary material for: Local Extinction and Unintentional Rewilding of Bighorn Sheep (Ovis canadensis) on a Desert Island
Source: PLoS One. 2014 Mar 19;9(3):e91358. doi: 10.1371/journal.pone.0091358 (PMC3960132; doi:10.1371/journal.pone.0091358)
Supplement: Table S1 — Primer sequences and fragment size. The genes sequenced or attempted from ancient fecal pellets from Tiburón Island, Mexico. (PDF) [file pone.0091358.s002.pdf]

Gene	Primer sequence	Fragment size (bp)
16S ribosomal RNA	F: AAG ACG AGA AGA CCC TAT GGA<br>R: CCG GTC TGA ACT CAG ATC ACG T	~350
12S ribosomal RNA	F: ATA CCG CCA TCT TCA GCA AA<br>R: CAT AGG TTA CAC CTT GAC CTA ACG	89
Control region, fragment 1	F: AGC GGG TTG TTG GTT TCA C<br>R: CCG TTC TAG TCA ACA TGC GTA	78
Control region, fragment 2	F: CGC ATG TTG ACT AGA ACG GAT T<br>R: CCA TGC ATA TAA GCA AGC ACA	104
Control region, fragment 3	F: ACA TTC AAG GTG GAC ATA GGG<br>R: ACA CGG ACT TTC CAC TCC AC	117
