# Supplementary material for: Local Extinction and Unintentional Rewilding of Bighorn Sheep (Ovis canadensis) on a Desert Island
Source: PLoS One. 2014 Mar 19;9(3):e91358. doi: 10.1371/journal.pone.0091358 (PMC3960132; doi:10.1371/journal.pone.0091358)
Supplement: Table S2 — Accession numbers of sequences used. The sequences used for primer development and sequence alignment for reference sequences and ancient fecal DNA of unknown origin from Tiburón Island, Mexico. (PDF) [file pone.0091358.s003.pdf]

Accession number	Species	Gene
JN632597.1	*Antilocapra americana*	mitochondrion, complete genome
JN632670.1	*Odocoileus hemionus*	mitochondrion, complete genome
JN632672.1	*Odocoileus virginianus*	mitochondrion, complete genome
FJ207535.1	*Oreamnos americanus*	mitochondrion, complete genome
JN181255.1	*Ovis canadensis canadensis*	mitochondrion, complete genome
NC_015889.1	*Ovis canadensis canadensis*	mitochondrion, complete genome
AF091706.1	*Antilocapra americana*	12S ribosomal RNA gene, partial sequence
OCU86983	*Ovis canadensis canadensis*	12S ribosomal RNA gene, partial sequence
AY091486	*Ovis canadensis canadensis*	control region, complete sequence
AY116621	[1]*Ovis canadensis mexicana*	control region, partial sequence, Tiburón-hap1
AY116622	[1]*Ovis canadensis mexicana*	control region, partial sequence, Tiburón-hap2
AY903995	*Ovis canadensis nelsoni*	control region, partial sequence, haplotype B
AY903996	*Ovis canadensis nelsoni*	control region, partial sequence, haplotype C
AY904011	*Ovis canadensis nelsoni*	control region, partial sequence, haplotype R
AY904013	*Ovis canadensis nelsoni*	control region, partial sequence, haplotype T
AY904015	*Ovis canadensis nelsoni*	control region, partial sequence, haplotype V
AY116623	[1]*Ovis canadensis weemsi*	control region, partial sequence

[1]Subspecies designations not warranted (*O. c. mexicana*) or probably not warranted (*O. c. weemsi*); should be considered *O. c. nelsoni*.
